# Cerebral vasospasm following aneurysmal subarachnoid hemorrhage: the impact of cocaine use, Hunt-Hess grade, and other risk factors

**DOI:** 10.1007/s00234-025-03713-y

**Published:** 2025-07-28

**Authors:** Aliana Rao, Samuel Ricci, Theodore Hannah, Erin Graves, Eric Quach, Kadir Erkmen, Rami Almefty

**Affiliations:** 1 Temple University, Philadelphia, PA USA; 2 Temple University Hospital, Philadelphia, PA USA

**Keywords:** Cerebral vasospasm, Aneurysmal SAH, Cocaine-induced vasospasm

## Abstract

**Introduction:**

Cerebral vasospasm (CV) is a common complication following aneurysmal subarachnoid hemorrhage (aSAH) that contributes to significant morbidity and mortality. While numerous identified CV risk factors exist, illicit substance use’s influence, particularly cocaine, remains controversial. This study aims to elucidate relationships between known risk factors and CV’s incidence, severity, and refractoriness.

**Methods:**

A retrospective chart review was conducted on all aSAH patients between 2014 and 2023 with inclusion criteria of confirmed aneurysms on digital subtraction angiography (DSA) and available urine drug screens (UDS). Demographic data, Hunt-Hess (HH), modified Fisher Scale (mFS), comorbidities, and vasospasm grades were collected. Outcomes, including vasospasm treatment counts, length of stay (LOS), and 3-month modified Rankin Scale (mRS) scores, were recorded.

**Results:**

Of 88 patients, 43% experienced CV. Cocaine use was significantly more prevalent in the CV group (26% vs. 8%, *p* = 0.04) and increased CV risk (OR = 4.11, 95% CI: 1.25–16.13, *p* = 0.03), independent of other factors. Higher HH grades were associated with increased CV incidence (OR = 1.75, *p* = 0.01), severity (β = 0.271, *p* = 0.02), and 3-month mRS scores (β = 0.81, *p* < 0.001). Female sex also predicted vasospasm incidence (OR = 4.78, *p* = 0.01). Older age was associated with worse long-term outcomes (β = 0.05, *p* = 0.004). In the multivariable analysis, cocaine revealed a significant increased risk of CV incidence (OR = 5.79, *p* = 0.02). Higher HH grades significantly impacted CV severity (OR = 0.30, *p* = 0.01) and worse long-term outcomes (OR = 0.957, *p* < 0.01) in the multivariable analysis.

**Conclusions:**

Females, those with positive cocaine use on UDS, and a history of T2DM were at an increased risk of CV with no impact on severity, recurrence, or 3-month outcomes. Older age was associated with worse long-term outcomes. HH grade was significantly associated with increased vasospasm severity, refractoriness to treatment, and worse long-term outcomes as per mRS scores.

**Supplementary Information:**

The online version contains supplementary material available at 10.1007/s00234-025-03713-y.

## Introduction

Cerebral vasospasm (CV) following spontaneous, aneurysmal subarachnoid hemorrhage (aSAH) is a well-described phenomenon, associated with substantial morbidity and mortality [1, 2]. CV tends to occur 3–14 days after the initial bleed and can lead to delayed cerebral ischemia (DCI) and infarction [3]. Despite the best medical treatment, approximately one-third of patients with CV suffer from persistent neurological deficits, due to decreased cerebral perfusion and DCI [3–5].

Multiple risk factors for CV have been posited. For example, females are 27% more likely to have CV as compared to their male counterparts [6]. A history of hypertension (HTN), both treated and untreated, has also been previously found to be an important risk factor for CV after aSAH as has a history of tobacco use [7, 8]. Furthermore, common scoring systems such as the Hunt Hess (HH) grade and modified Fisher Scale (mFS) scores have also been found to correlate with CV occurrence [7, 9, 10]. More recently, other factors have been identified as possibly protective in nature against CV risk, including older age [6, 11].

Pre-morbid substance abuse, particularly the use of cocaine, is common among aSAH patients. Given the vasoactive properties of cocaine, it was postulated to increase the risk of vasospasm in aSAH patients [12]. There are several studies supporting this theory. In 2001, Conway et al. compared 27 aSAH patients recent cocaine exposure and found an increased risk of CV compared to controls, though with no effect on clinical outcomes [13]. Howington et al. found cocaine use increased the risk of vasospasm by almost 3 fold [14]. In a large meta-analysis, Florez-Perdomo et al. found that cocaine use was associated with increased risk of vasospasm and higher mortality [15]. Conversely, Alaraj et al. came to the opposite conclusion in 2010, finding no difference in both incidence of symptomatic vasospasm or clinical outcome between 31 cocaine users with aSAH and controls [16]. Despite the numerous evaluations of the effect of cocaine on vasospasm in SAH patients, there has been no evaluation of whether cocaine induces more severe or more refractory vasospasm than that experienced by other patients with aSAH.

The present study aims to evaluate the effect of cocaine on aSAH vasospasm incidence, severity, and refractoriness to treatment.

## Methods

### Patient population

A retrospective chart review was conducted on all patients admitted with non-traumatic SAH at Temple University Hospital between January 2014 and December 2023. The hospital’s Institutional Review Board (IRB) approved the study. All patients with a confirmed aneurysm on digital subtraction angiography (DSA) and an admission urine drug screen (UDS) available for review were included in analyses. UDS included data on fentanyl, amphetamines, barbiturates, buprenorphine, cannabinoids, cocaine, fentanyl, oxycodone, opiates, and phencyclidine, and buprenorphine use. Hospital course information was documented, including the date of SAH and primary aneurysm treatment, days of nimodipine administration, and overall length of stay (LOS). mFS and HH grades were included for each aSAH. Demographic data including age, sex, race, and past medical history was collected. Past medical history included hypertension (HTN), hyperlipidemia (HLD), type 2 diabetes mellitus (T2DM), heart failure (HF), and chronic kidney disease (CKD) as well as body-mass index (BMI) measurements. Exclusion criteria included age under 18, SAH from arteriovenous malformation (AVM) or other non-aneurysmal vascular lesion, pregnancy, and incarceration.

### Cohorts

The patients were divided into cohorts based on the presence or absence of vasospasm during the patient’s admission for aSAH.

### SAH grading

Each patient’s HH grade was determined at the time of presentation to the emergency department. The mFS was used to classify the severity of aSAH on the patient’s presenting CT scan.

### Vasospasm severity grading

All patients underwent repeat DSA 7 days after initial SAH bleed to evaluate for vasospasm. DSA was evaluated by residents, fellows, and attendings for evidence of radiographic vasospasm. Each patient’s angiographic imaging was reviewed by an attending and an endovascular fellow to determine the vasospasm grade. Any disagreements were resolved by discussion or evaluation by another attending. The evaluators did not have access to the patient’s chart while reviewing the imaging, but the patient’s name could not be hidden from the imaging. However, the cases were conducted over a 10-year period, the evaluation of the imaging was not done until six months to a year after the final case, and the physicians conducting the review of imaging were not involved in most of the cases. The possibility of bias in vasospasm grading from prior knowledge of patient history is low, but not zero. 

**Fig. 1:**
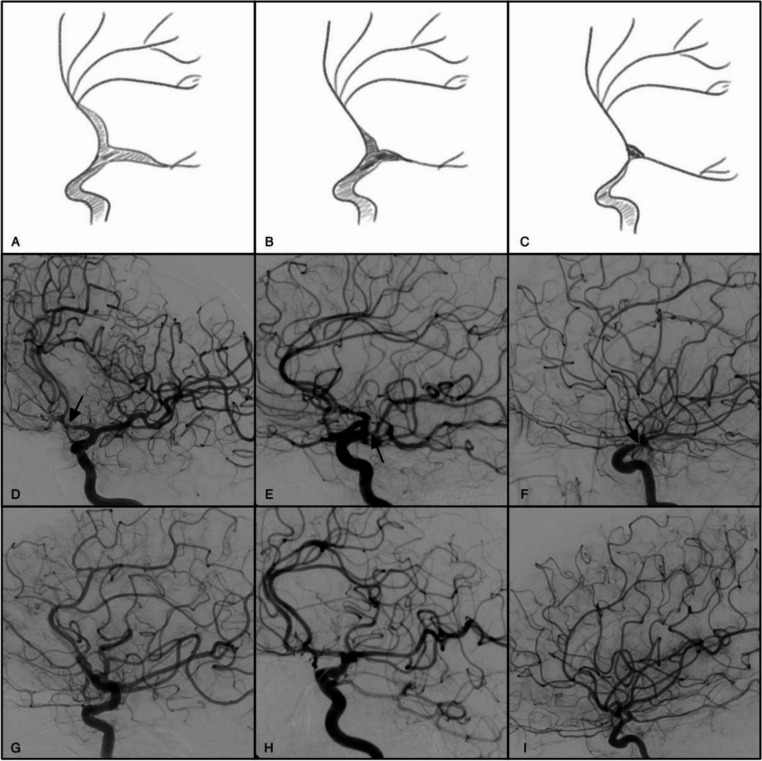
(**A**-**C**) demonstrates drawings of vasospasm grades 1, 2, and 3. (**D**-**F**) demonstrates DSA images revealing grades 1, 2, and 3 (as shown by arrows) respectively, and correlates with the drawing. (**G**-**I**) is DSA 6 months post vasospasm incidence with a grade of 0

DSAs were reviewed and CV was graded on a scale of 0 to 3 using the Visual Cerebral Vasospasm Classification, as described by Merkel et al. [17]. Grade 0 is classified as all vessels showing physiologic shape. Grade 1 is CV in A1, A2 & M2. Grade 2 is CV in M1 & ICA terminus. Grade 3 is classified as CV causing severe reduction in ICA, A1 & M1. Figure 1 serves as a reference for vasospasm grading on initial presentation. The number of angiograms performed for vasospasm treatment was also recorded. All patients at our institution receive nimodipine for vasospasm prophylaxis and systolic blood pressure is liberalized to 180-200 depending on the clinical scenario. If vasospasm was identified on DSA, patients were treated with intra-arterial verapamil first, then followed with intra-arterial nitroglycerin, or less frequently, milrinone. If the vasospasm did not resolve after delivery of the pharmacologic agents or recurred, balloon angioplasty was utilized.

### Long term outcomes

Long-term outcomes were measured using modified Rankin Scale (mRS) scores based on functionality at clinical follow-ups. mRS scores were assessed at 3 months.

### Data analysis

In this study, statistical analyses were performed using R software (version 2023.12.1). Data were imported from an Excel file and prepared by converting categorical variables (e.g., vasospasm status, sex, race, hypertension, diabetes) into factors and ensuring continuous variables (e.g., age, BMI) were numeric. Descriptive statistics were calculated, with continuous variables summarized using medians and standard deviations or standard errors, and categorical variables summarized using counts and percentages. Comparative analyses between patients with and without vasospasm were conducted using independent two-sample *t*-tests for continuous variables (age, BMI, HH grade, mFS, LOS, total nimodipine dosage, and 3-month mRS scores) and chi-square tests for categorical variables (sex, race, hypertension, hyperlipidemia, diabetes, chronic kidney disease, cocaine use, tobacco use, and aneurysm location). Logistic regression models were employed to assess the association between vasospasm incidence and patient factors—both univariate and multivariable analyses included sex, HTN, substance use, and tobacco use variables. Linear regression analyses were conducted to evaluate relationships between these factors and vasospasm severity, as well as vasospasm refractoriness, adjusting for potential confounders.

To mitigate the risk of overfitting, we employed a five-fold cross-validation approach (trainControl method in the *caret* package), whereby the dataset was partitioned into five subsets; each subset was iteratively used as a validation set while the remaining subsets formed the training data. We evaluated model performance using cross-validation RMSE and R-squared for continuous outcomes (linear regression) and classification metrics (e.g., accuracy) for binary outcomes (logistic regression). Multicollinearity among predictors was assessed using variance inflation factors (VIF), and any variables exhibiting excessive collinearity (VIF > 5) were considered for removal from the final models. Results were reported with odds ratios or regression coefficients, 95% confidence intervals, and corresponding *p*-values, with statistical significance set at *p* < 0.05.

## Results

### Demographics

The mean age of patients in both the no-vasospasm and vasospasm groups was comparable (55 vs. 53 years, *p* = 0.97). However, there was a significant difference was observed in sex distribution, with females representing 89.5% of the vasospasm group compared to 64% in the no vasospasm group (*p* = 0.01). Cocaine use was significantly more common in the vasospasm group (26% vs. 8%, *p* = 0.04).

Racial distribution did not differ significantly between groups (*p* = 0.78), with Black and Hispanic patients being predominant in both. There was also no significant difference in most comorbidities, including hypertension, hyperlipidemia, diabetes mellitus, and chronic kidney disease. Tobacco use (both current and ever) did not differ between groups. BMI was similar in both groups, with no significant difference (27 vs. 29 kg/m², *p* = 0.60). Neurological status, measured by the HH grade, was significantly higher in the vasospasm group (3 vs. 2, *p* = 0.01), but did not reach significance in the mFS (*p* = 0.057).

Patients with vasospasm had a significantly longer length of stay (14 vs. 21 days, *p* = 0.0001). Primary aneurysm location was not significantly different (*p* = 0.25). The 3-month mRS scores were not significantly different between groups (*p* = 0.94). (Table 1)

**Table 1:** Demographic and operative details

Variable			No Vasospasm	Vasospasm	T-Value	Chi Value	*p*-value
			*n* = 50 (57%)	*n* = 38 (43%)			
Age (mean)					−0.03		0.98*
	Mean		55	53			
	Standard Error of Mean		2	2			
Sex						6.18	**0.013**
	Female		32 (64%)	34 (89.5%)			
	Male		18 (36%)	4 (10.5%)			
Race						1.74	0.78
	Black		25 (50%)	21 (55%)			
	Hispanic		14 (28%)	10 (26%)			
	White		7 (14%)	5 (13%)			
	Asian		2 (4%)	0 (0%)			
	Unknown		2 (4%)	2 (5%)			
Comorbidities							
	Hypertension		35 (74%)	24 (65%)		0.51	0.47
	Hyperlipidemia		11(23%)	7 (19%)		0.05	0.81
	Heart Failure		4 (8%)	0 (0%)		1.69	0.19
	Diabetes Mellitus		14 (30%)	4 (11%)		3.37	0.06
	Chronic Kidney Disease		1 (2%)	0 (0%)		0	1
	BMI (kg/m^3)		27 ± 7	29 ± 6	−0.53		0.59*
Substances							
	Cocaine		4 (8%)	10 (26%)		4.13	**0.04**
	Amphetamines		2 (4%)	1 (2.6%)		0	1
	Opioids		9 (18%)	10 (26%)		0.46	0.499
	Fentanyl		4 (8%)	5 (13%)		0.19	0.66
	Cannabis		13 (26%)	9 (24%)		0	1
	Tobacco (Current)		25 (50%)	23 (40%)		0.58	0.44
	Tobacco (Ever)		36 (72%)	25 (65%)		1.54	0.69
Hunt Hess			2 ± 0.96	3 ± 1.05	−2.57		0.01*
Modified Fisher Score			3 ± 1.04	3 ± 0.654	−1.93		0.06*
Length of Stay			14 ± 9.15	21 ± 9.67	−3.92		< 0.01*
Days on Nimodipine			10 ± 6.73	9.5 ± 7.92	0.91		0.37
Primary Aneurysm						4.12	0.25
	ACA		10 (20%)	15 (39%)			
	ICA		18 (36%)	8 (26%)			
	MCA		13 (26%)	7 (18%)			
	Posterior Circulation		9 (18%)	6 (15%)			
3 m mRS			1 ± 1.92	2 ± 1.58	0.07		0.94*

Vasospasms were characterized as Grade 1, 2, or 3 with 18%, 42%, and 34% of graded vasospasms respectively. Number of vasospasm treatments varied, with 2 treatments as the mode. Time from SAH to vasospasm was 6.5 days with an SD of 2 days (Table 2).

**Table 2:** Vasospasm characteristics

Vasospasm Severity		
	Grade I	7 (18%)
	Grade II	16 (42%)
	Grade III	14 (34%)
Number of Vasospasm Treatments		
	1	8 (21%)
	2	17 (44%)
	3	5 (13%)
	4+	6 (16%)
	Unknown	2 (0.5%)
Time from SAH to Vasospasm (Days)		6.5 ± 2

### Single variable logistic regression for vasospasm incidence

We then analyzed the single variable logistic regression results for vasospasm incidence to explore potential predictors of this outcome (Table 3). Among the variables, sex demonstrated a significant association (OR = 4.78, 95% CI: 1.58–17.93, *p* = 0.0097). HH also showed significance (OR = 1.75, 95% CI: 1.14–2.78, *p* = 0.0137), suggesting a measurable impact on vasospasm incidence. In addition, T2DM reached significance (OR = 0.29, 95% CI: 0.07–0.89, *p* = 0.0427). Meanwhile, the mFS had a near-significant p-value (OR = 1.59, 95% CI: 0.97–2.74, *p* = 0.0773), and the location of the primary aneurysm was also near-significant (OR = 0.37, 95% CI: 0.12–1.11, *p* = 0.0802). All other variables did not exhibit p-values below the 0.05 threshold, suggesting they were not significant in this context. Overall, the results highlight three significant predictors (sex, HH, and T2DM) and two near-significant factors (mFS and Primary Aneurysm), while the remaining variables did not show notable associations.

**Table 3:** Single-variable logistic regression for vasospasm incidence and recurrence

	Vasospasm Incidence			Vasospasm Recurrence			
Variable	OR	CI	*p*	OR	CI	*p*	
Age	1.001	0.965–1.038	0.976	0.98	0.901–1.064	0.62	
Sex (Female)	4.781	1.584–17.925	**0.01**	0	NA - NA	0.994	
HH	1.745	1.137–2.776	**0.014**	2.322	1.047–6.026	**0.052**	
mFS	1.587	0.971–2.740	0.077	1.66	0.377–8.501	0.508	
Pri Aneurysm	0.37	0.118–1.107	0.08	0.107	0.005–0.910	0.067	
Tobacco (E)	0.748	0.299–1.871	0.532	1.5	0.259–7.722	0.63	
Tobacco (C)	0.652	0.274–1.525	0.327	1.25	0.254–7.074	0.787	
Amphetamine	0.649	0.029–7.022	0.728	0	NA - NA	0.994	
Cocaine	4.107	1.245–16.137	**0.027**	0.556	0.105–3.258	0.49	
Opiates	1.627	0.584–4.598	0.35	1	0.178–7.894	1	
Fentanyl	1.742	0.430–7.511	0.433	0.36	0.048–3.185	0.316	
Cannabis	0.883	0.324–2.336	0.804	0.217	0.037–1.187	0.077	
Hypertension	0.633	0.244–1.624	0.341	0.515	0.067–2.723	0.463	
Hyperlipidemia	0.764	0.253–2.185	0.619	0.5	0.076–4.213	0.479	
T2DM	0.286	0.075–0.892	**0.043**	0.84	0.090–18.451	0.887	
BMI	1.018	0.951–1.092	0.601	1.007	0.880–1.174	0.925	

### Single variable logistic regression for vasospasm recurrence

Following our analysis of factors driving vasospasm incidence, we sought to understand the drivers influencing repeated episodes of vasospasm (Table 3). In these analyses, HH approached significance (OR = 2.32, 95% CI: 1.05–6.03, *p* = 0.0524), suggesting it may be an important factor, though it narrowly missed a conventional significance level of *p* < 0.05. The location of the primary aneurysm was also near significant (OR = 0.11, 95% CI: 0.00–0.91, *p* = 0.0671), indicating a potential protective relationship. Cannabis use followed a similar pattern (OR = 0.22, 95% CI: 0.04–1.19, *p* = 0.0768), showing a trend toward significance. Age, sex, and other variables did not meet or approach significance, indicating no strong evidence for their influence on recurrence in this single-variable context. HH, primary aneurysm location, and cannabis emerged as variables of interest, despite not achieving full statistical significance. All other variables were clearly non-significant.

### Single variable linear regression for maximum recorded vasospasm severity

Our next analysis addressed the variables contributing to recorded vasospasm severity, specifically, the maximum recorded vasospasm severity (Table 4). HH was significant (estimate = 0.27, 95% CI: 0.05–0.50, *p* = 0.0197), indicating a positive association with the severity measure. A history of previous tobacco use (Tobacco (E)) showed a borderline p-value, hinting at near significance (estimate = 0.50, 95% CI: −0.02–1.02, *p* = 0.0586). Apart from these two, all other variables, such as age, sex, and BMI, demonstrated p-values above 0.05, suggesting they were not strongly related to maximum vasospasm severity in this univariate context. Therefore, HH emerged as the primary significant predictor, with previous history of tobacco use remaining an interesting but inconclusive factor.

**Table 4:** Single-variable logistic regression for maximum vasospasm and 3-month mRS

	Maximum Recorded Vasospasm			3-month mRS			
Variable	β	CI	*p*	β	CI	*p*	
Age	−0.016	−0.041–0.010	0.225	0.050	0.016–0.084	**0.004**	
Sex (Female)	−0.182	−1.096–0.732	0.689	0.252	−0.729–1.232	0.610	
HH	0.271	0.046–0.496	**0.020**	0.805	0.445–1.165	**0.000**	
mFS	−0.016	−0.489–0.457	0.945	0.379	−0.097–0.856	0.116	
Pri Aneurysm	0.100	−0.498–0.698	0.735	−1.077	−2.182–0.027	**0.056**	
Tobacco (E)	0.502	−0.019–1.023	**0.059**	−0.075	−1.001–0.850	0.871	
Tobacco (C)	0.400	−0.094–0.894	0.109	−0.598	−1.449–0.252	0.165	
Amphetamine	−0.171	−1.711–1.368	0.822	−1.679	−4.217–0.859	0.191	
Cocaine	−0.231	−0.790–0.329	0.408	−0.006	−1.353–1.341	0.993	
Opiates	−0.222	−0.802–0.357	0.441	−0.848	−1.873–0.177	0.103	
Fentanyl	0.271	−0.455–0.997	0.453	−1.238	−2.739–0.262	0.104	
Cannabis	−0.074	−0.658–0.510	0.798	−0.017	−1.018–0.984	0.973	
Hypertension	−0.221	−0.742–0.301	0.396	0.320	−0.613–1.254	0.496	
Hyperlipidemia	−0.400	−1.065–0.265	0.230	−0.096	−1.124–0.932	0.852	
T2DM	0.094	−0.711–0.899	0.814	0.313	−0.690–1.315	0.536	
BMI	0.005	−0.041–0.050	0.838	0.013	−0.067–0.093	0.739	

### Single variable linear regression for three-month modified Rankin score

We then analyzed the single variable linear regression results for the three-month modified Rankin score to see which factors might correlate with functional outcomes (Table 4). Age was statistically significant (estimate = 0.05, 95% CI: 0.02–0.08, *p* = 0.0043), indicating that higher age could be associated with increased scores. HH was also significant (estimate = 0.80, 95% CI: 0.45–1.16, *p* < 0.0001), suggesting a strong relationship between HH grading and worse mRS scores. The location of the primary aneurysm had a near-significant effect (estimate = −1.08, 95% CI: −2.18–0.03, *p* = 0.0558), hinting at a possible protective factor but not reaching conventional significance. Other variables, such as sex and mFS, did not meet the *p* < 0.05 cutoff, indicating they were less influential on the three-month mRS outcome in this univariate analysis. Thus, age and HH emerged as clear predictors in this model, with the location of the primary aneurysm deserving further investigation. All remaining variables demonstrated nonsignificant p-values.

### Multivariable logistic regression for vasospasm incidence

In this cohort, vasospasm occurred with significant associations to patient sex and cocaine use Multivariable logistic regression identified female sex as a strong independent predictor, increasing the odds of vasospasm by over fourfold (OR 4.08, 95% CI 1.23–16.74, adjusted *p* = 0.042). Similarly, cocaine use was associated with a comparable increase in vasospasm risk (OR 4.35, 95% CI 1.22–18.73, adjusted *p* = 0.031). While Hunt-Hess grade demonstrated a positive trend (OR 1.55, 95% CI 0.97–2.54, *p* = 0.07), it did not reach statistical significance after adjustment (*p* = 0.074). Assessment for multicollinearity revealed low variance inflation factors (all < 1.05), indicating independent effects of each predictor within the model. The model’s internal validity was confirmed using five-fold cross-validation, which demonstrated consistent predictive performance without evidence of overfitting.

### Multivariable logistic regression for vasospasm recurrence

Multivariable analysis examining factors associated with vasospasm recurrence revealed no statistically significant predictors among sex, HH grade, or cocaine use. HH grade approached significance with an odds ratio suggesting more than a twofold increased risk of recurrence (OR 2.32, 95% CI 1.04–6.15, *p* = 0.057), but this did not hold after multiple testing correction (adjusted *p* = 0.23). Both sex and cocaine use showed no significant effect on recurrence rates (adjusted *p* > 0.05). Variance inflation factors remained near 1.0 across variables, confirming the absence of multicollinearity. Internal validation through five-fold cross-validation supported the stability and reliability of the model despite the lack of significant predictors for vasospasm recurrence (Table 5).

**Table 5:** Multi-variable logistic regression results for vasospasm incidence and recurrence

	Vasospasm Incidence			Vasospasm Recurrence			
Variable	OR	CI	*p*	OR	CI		*P*-Value
Cocaine	4.350	1.218–18.73	**0.031**	0.498	0.076–3.365		0.457
Sex (F)	4.084	1.230–16.74	**0.031**	0.000	NA - NA		0.993
Hunt Hess	1.545	0.967–2.535	0.074	2.315	1.037–6.149		0.057

### Multivariable linear regression for maximum recorded vasospasm severity

The question of vasospasm severity remains unexplored in model accounting for other significant variables (Table 6). In this adjusted model, the HH again showed a significant positive association (estimate = 0.30, 95% CI: 0.08–0.52, *p* = 0.010). Tobacco use at the time of admission (Tobacco (C)) emerged as near significant (estimate = 0.48, 95% CI: −0.09–1.04, *p* = 0.096), suggesting a possible trend but not fully meeting the conventional cutoff. Other variables, including cocaine, HTN, sex, and prior history of tobacco use, exhibited nonsignificant p-values. Assessment of VIF values revealed a range from 1.03 to 1.62, indicating relatively low levels of multicollinearity among the predictors. This result implies that individual regression coefficients were not inflated due to correlation between predictors. Ultimately, these findings highlight HH as a consistent predictor of maximum vasospasm severity, with tobacco use continuing to show a marginal effect.

**Table 6:** Multiple-variable logistic regression for maximum vasospasm and 3-month mRS

	Maximum Recorded Vasospasm			Three Month mRS			
Variable	OR	CI	p	OR	CI		p
Cocaine	−0.294	−0.792–0.205	0.238	0.7708	0.235–2.527		0.6604
Hypertension	−0.345	−0.811–0.121	0.141	1.158	0.518–2.584		0.7167
Hunt Hess	0.300	0.078–0.523	**0.010**	2.778	1.879–4.111		**< 0.001**
Sex (F)	0.035	−0.778–0.849	0.930	0.7194	0.2852–1.811		0.4776
Tobacco (C)	0.477	−0.09–1.045	0.096	0.3751	0.144–0.9727		**0.0439**
Tobacco (E)	0.264	−0.336–0.864	0.376	1.111	0.3916–3.153		0.8398
Vasospasm	-	-	-	0.524	0.2306–1.194		0.1217

### Multivariable linear regression for three-month modified Rankin score

At three months, multivariable linear regression identified HH score as a significant independent predictor of mRS outcomes (estimate 1.022, 95% CI 0.631 to 1.412, adjusted *p* = 1.88 × 10^−5). Other variables including sex (estimate − 0.330, 95% CI −1.253 to 0.593, adjusted *p* = 0.83), cocaine use (estimate − 0.261, 95% CI −1.445 to 0.923, adjusted *p* = 0.83), current tobacco use (estimate − 0.981, 95% CI −1.935 to −0.028, adjusted *p* = 0.176), past tobacco use (estimate 0.106, 95% CI −0.937 to 1.148, adjusted *p* = 0.84), hypertension (HTN; estimate 0.146, 95% CI −0.657 to 0.949, adjusted *p* = 0.83), and presence of vasospasm (Spasm; estimate − 0.646, 95% CI −1.470 to 0.177, adjusted *p* = 0.32) did not reach statistical significance after multiple testing correction. Multicollinearity was low across predictors, with variance inflation factors ranging from 1.06 to 1.88.

## Discussion

The principal aim of this study was to determine the impact of various drugs, measured via a UDS at the time of admission, and other risk factors for vasospasm on the vasospasm incidence, severity, and refractory nature in patients with aneurysmal subarachnoid hemorrhage. Our findings demonstrate that female sex, higher HH scores, cocaine use, and T2DM at the time of hospital admission were strongly associated with an increased incidence of vasospasm. All other substances analyzed (amphetamines, opiates, fentanyl, and cannabis) were not associated with increased vasospasm incidence, severity, refractoriness, nor three-month mRS, with the exception of cannabis approaching significance for vasospasm recurrence. However, cocaine, sex, and T2DM were not significantly correlated with the severity of vasospasm nor recurrence. Notably, HH grades were significantly correlated with refractory vasospasm and vasospasm severity. Additionally, HH grades will worsen three-month outcomes as measured via mRS scores. Furthermore, cocaine was the only modifiable risk factor associated with any of the outcomes evaluated in this study.

The relationship between cocaine use and cerebral vasospasm has been increasingly recognized in recent years, with several studies highlighting the significant risks and clinical challenges associated with this condition. One study reported two cases of chronic cocaine users who experienced severe vasospasm during neurointerventional procedures, underscoring the heightened risk of iatrogenic vasospasm in this patient group [18]. Another case report detailed the development of delayed cerebral ischemia and subsequent cortical blindness in a patient with a history of cocaine use, exacerbated by repeated cocaine use weeks after an aSAH [19]. Additionally, a large inpatient study identified cocaine use as a significant risk factor for the development of cerebral vasospasm following traumatic intracranial hemorrhage, further highlighting the dangers associated with this substance [20]. A comprehensive review discussed the mechanisms by which cocaine can lead to both ischemic and hemorrhagic strokes, emphasizing vasospasm as a primary mechanism of injury [21]. Lastly, a small case series focused on the outcomes of mechanical thrombectomy in patients with large vessel occlusions related to cocaine use, highlighting the role of vasospasm in contributing to poor outcomes in these cases [22]. These studies collectively underscore the critical impact of cocaine use on cerebral vasospasm, illustrating the complex interplay between substance abuse and cerebrovascular pathology. Of studies looking specifically at the impact of cocaine use on the incidence of CV following aSAH, our findings were consistent with the findings throughout most of the literature showing an elevated risk of CV specifically in patients with recent cocaine use. Similar to some prior research, we too found no impact on the long-term clinical outcomes in these patients when compared to controls [13, 23]. We additionally looked at the severity of the CV and the degree of refractoriness to treatment, as indicated by the number of repeated treatments required, and found no difference between those with premorbid substance use and those without.

The occurrence of CV contributes to prolonged LOS, likely due to its association with complications and the need for additional treatments and extended monitoring [23]. In patients with no evidence of radiographic vasospasm within 5–7 days of SAH, hospital stays were notably shorter [24]. Our data confirms that LOS was increased in patients with at least one incidence of vasospasm. Due to the monitoring efforts required, such as transcranial Doppler, and further treatment paradigms of intra-arterial vasodilators or balloon angioplasty, longer hospital stays are not unreasonable in patients with symptomatic vasospasm [25].

Although there is abundant research supporting higher HH grades impacting vasospasm incidence, there exists limited data on the impact of HH grades on vasospasm severity and recurrence [6, 7, 23]. Our findings reveal an association between HH grades ≥ 3 and increased vasospasm severity with no impact of HH grades on CV incidence. Previous studies show HH grades of 4 and 5 are increasingly associated with poorer long-term outcomes [26, 27]. Our data reveals higher HH grades, specifically 4 and 5, are associated with worse long-term functional outcomes and more deficits at the 3-month mark, which supports earlier findings.

Sex remained a positive predictor of vasospasm incidence in the single-variable analysis, and approaches significance in the multivariable logistic regression analysis. Further investigation into the relationship between female sex and vasospasm incidence revealed no significant differences when controlling for age or race. The relationship between female sex and vasospasm has been explored across various clinical contexts, revealing significant associations that may be influenced by hormonal and age-related factors. Conversely, in cases of aSAH, female sex has been identified as a significant predictor of symptomatic vasospasm, with women, particularly those aged 55 to 74, being at higher risk for vasospasm-related complications; this heightened risk is likely due to hormonal changes associated with menopause [28]. The influence of female sex hormones on vasospasm is further supported by studies on conditions like Raynaud’s phenomenon, although patient-reported data does not consistently indicate a strong hormonal impact on vasospastic episodes [29]. Furthermore, women exhibit greater sensitivity to coronary vasomotor dysfunction, including both coronary microvascular dysfunction and epicardial vasospasm, often at lower doses of acetylcholine compared to males [30]. These findings underscore the complex interplay between sex, hormonal status, and vascular health, highlighting the need for further research to understand the mechanisms driving these sex-specific differences in incidence and severity.

Race-related differences in the clinical outcomes of aSAH patients are well documented. Minority populations, such as Black and Hispanic, have a higher incidence of aSAH than white patients [31]. We did not find race to be a prognostic factor for clinical outcomes after aSAH. Rosen et al. 2005 postulates that the higher mortality rate among Black patient populations with aSAH is in part due to the increased incidence of aSAH in that population and not due to race serving as a prognostic factor [32]. Similarly, other studies have found that while minority populations may have comorbidities, such as increased blood pressure, they do not necessarily translate to poorer outcomes in aSAH [32, 33].

Age did not emerge as a meaningful predictor of vasospasm incidence in our single-variable logistic models (OR ≈ 1.00, *p* ≈ 0.98). However, in the linear regression examining three-month functional outcomes, increasing age was significantly associated with higher (i.e., worse) mRS scores (*p* = 0.004), a finding consistent with prior literature indicating poorer long-term recovery among older patients [20]. Interestingly, T2DM demonstrated a statistically significant protective association against vasospasm in unadjusted analyses (OR ≈ 0.29, *p* = 0.043), but this effect was no longer significant in multivariable models. One possibility is that T2DM status may coexist with other risk factors or clinical management practices (e.g., tighter glucose control, closer monitoring) that confound its relationship with vasospasm risk. Overall, our results suggest that while advanced age may worsen long-term functional outcomes, the impact of T2DM on vasospasm remains uncertain once potential confounders are taken into account.

### Limitations

This study has several limitations that must be acknowledged. Firstly, the retrospective nature of the study limits the ability to establish causality between vasospasm incidence and any of the risk factors analyzed. Additionally, the reliance on chart reviews and the availability of admission urine drug screens (UDS) may have introduced selection bias, as only patients with documented UDS were included in the analysis. The sample size, while sufficient for initial analyses, may not be large enough to detect more subtle associations or to generalize the findings to broader populations. Furthermore, the study did not account for potential confounding variables such as the use of other vasoactive substances, antiplatelet therapy use at the time of admission, variations in treatment protocols, or the timing of cocaine use relative to the onset of vasospasm. Lastly, the study’s single-center design limits the generalizability of the results, and the findings may not apply to other institutions with different patient populations or treatment approaches. Future studies should aim to address these limitations by utilizing prospective, multicenter designs with larger sample sizes and more comprehensive data collection.

## Conclusion

Our study demonstrates that of patients with aSAH, females, those with positive cocaine use on UDS, and history of T2DM were at an increased risk of CV with no impact on severity, recurrence, or 3-month outcomes. Older age was associated with worse long-term outcomes. HH grade was significantly associated with increased vasospasm severity, refractoriness to treatment, and worse long-term outcomes as per mRS scores.

## Supplementary Information

Below is the link to the electronic supplementary material.

ESM 1 (DOCX 15.9 KB)

## Data Availability

Data is provided within the manuscript or supplementary information files.
