# Supplementary material for: Cerebral vasospasm following aneurysmal subarachnoid hemorrhage: the impact of cocaine use, Hunt-Hess grade, and other risk factors
Source: Neuroradiology. 2025 Jul 28;67(9):2337–47. doi: 10.1007/s00234-025-03713-y (PMC12546373; doi:10.1007/s00234-025-03713-y)
Supplement: Supplementary file 1 — (DOCX 15.9 KB) [file 234_2025_3713_MOESM1_ESM.docx]

**Supplemental Table 1** Table showing the comparison of clinical and demographic variables between patients with Mild Hunt-Hess (HH) scores (N = 45) and Severe HH scores (N = 43). Continuous variables (Age, modified Fisher Scale [mFS], Body Mass Index [BMI]) are presented as mean ± standard deviation and compared using two-sample t-tests. Categorical variables are presented as counts with percentages and compared using chi-square tests. Statistically significant differences were observed in mFS (p = 0.0016), sex distribution favoring females in the Severe HH group (p = 0.0363), and diabetes mellitus prevalence (p = 0.0226). No significant differences were found in race, primary vascular territory, tobacco use, cocaine, amphetamine, opiate, fentanyl, cannabis use, hypertension, hyperlipidemia, heart failure, or chronic kidney disease. These analyses support an independent association between HH severity and select clinical variables, suggesting potential direct relationships influencing disease severity and outcomes.

| **Variable** | **Mild HH  N = 45** | **Severe HH  N = 43** | **Statistic** | **p-value** |
| --- | --- | --- | --- | --- |
| Age | 52.6 ± 13.4 | 55.9 ± 9.8 | -1.326 | 0.1887 |
| mFS | 2.8 ± 1.08 | 3.4 ± 0.54 | -3.294 | **0.0016** |
| BMI | 28.2 ± 6.72 | 28.9 ± 6.27 | -0.522 | 0.6032 |
| Sex (F) | 29 (64.4%) | 37 (86.0%) | 4.381 | **0.0363** |
| Race |  |  | 3.638 | 0.4573 |
| Asian | — | 2 (4.7%) |  |  |
| Black | 25 (55.6%) | 21 (48.8%) |  |  |
| Hispanic | 12 (26.7%) | 12 (27.9%) |  |  |
| Unknown | 3 (6.7%) | 1 (2.3%) |  |  |
| White | 5 (11.1%) | 7 (16.3%) |  |  |
| Primary |  |  | 1.898 | 0.5938 |
| ACA | 10 (22.2%) | 15 (34.9%) |  |  |
| ICA | 15 (33.3%) | 13 (30.2%) |  |  |
| MCA | 11 (24.4%) | 9 (20.9%) |  |  |
| POST | 9 (20.0%) | 6 (14.0%) |  |  |
| Tobacco (Ever) | 31 (68.9%) | 30 (69.8%) | 0 | 1 |
| Tobacco (Current) | 20 (44.4%) | 20 (46.5%) | 0 | 1 |
| Cocaine | 5 (11.1%) | 9 (20.9%) | 0.936 | 0.3334 |
| Amphetamine | 1 (2.2%) | 2 (4.7%) | 0 | 0.968 |
| Opiates | 13 (28.9%) | 6 (14.0%) | 2.082 | 0.149 |
| Fentanyl | 5 (11.1%) | 4 (9.3%) | 0 | 1 |
| Cannabis | 12 (26.7%) | 10 (23.3%) | 0 | 0.902 |
| Hypertension | 30 (69.8%) | 29 (70.7%) | 0 | 1 |
| Hyperlipidemia | 10 (23.3%) | 8 (19.5%) | 0 | 0.8792 |
| Heart Failure | 3 (7.0%) | 1 (2.4%) | 0 | 0.6429 |
| Diabetes Mellitus | 14 (32.6%) | 4 (9.8%) | 5.198 | **0.0226** |
| Chronic Kidney Disease | 1 (2.3%) | 0 (0%) | 0 | 1 |
